# Kidney–Lung Crosstalk in Acute Nephrologic Involvement: Mechanisms, Complement Activation, and Implications for Multiorgan Dysfunction

**DOI:** 10.3390/life16020276

**Published:** 2026-02-05

**Authors:** Giuliana Martino, Francesca Tinti, Marco Alfonso Perrone, Stefano Condò, Veronica Castagnola, Simone Manca de Villahermosa, Paola Triggianese, Marzena Olesinska, Alessandra Valentini, Sergio Bernardini, David Della Morte, Ferdinando Iellamo, Luca Salomone, Silvia Lai, Anna Paola Mitterhofer

**Affiliations:** 1 Nephrology and Dialysis Unit, University Hospital Tor Vergata, 00133 Rome, Italysimone.manca@ptvonline.it (S.M.d.V.); annapaola.mitter@uniroma2.it (A.P.M.); 2 Department of Clinical and Molecular Medicine, Nephrology and Dialysis Unit, University Hospital S. Andrea, Sapienza University of Rome, 00189 Rome, Italy; 3 Department of Clinical Sciences and Translational Medicine, University of Rome Tor Vergata, 00133 Rome, Italyiellamo@uniroma2.it (F.I.); 4 Department of Systems Medicine, University of Rome Tor Vergata, 00133 Rome, Italy; 5 Department of Biomedicine and Prevention, University of Rome Tor Vergata, 00133 Rome, Italydavid.dellamorte@uniroma2.it (D.D.M.); 6 Department of Connective Tissue Diseases, National Institute of Geriatrics, Rheumatology and Rehabilitation, 02-637 Warsaw, Poland; 7 Clinical Biochemistry Unit, University Hospital Tor Vergata, 00133 Rome, Italybernards@uniroma2.it (S.B.); 8 Department of Experimental Medicine, University of Rome Tor Vergata, 00133 Rome, Italy; 9 Department of Biology and Prevention, University of Rome Tor Vergata, 00133 Rome, Italy; 10 Department of Translational and Precision Medicine, Nephrology Unit, Umberto I University Hospital, Sapienza University of Rome, 00161 Rome, Italy

**Keywords:** kidney–lung crosstalk, acute kidney injury (AKI), acute lung injury (ALI), complement system, C5a–C5aR1 signaling, NETosis, multiorgan dysfunction, ischemia–reperfusion injury, complosome

## Abstract

Acute kidney injury (AKI) is a systemic syndrome capable of inducing remote organ dysfunction. Kidney–lung crosstalk is a form of interorgan communication in acute nephrology, with the heart acting as a pivotal intermediary. Emerging evidence supports the involvement of a gut–lung–kidney axis. Complement activation in these multiorgan crosstalk has emerged as a central amplifier of multiorgan damage. We reviewed the literature on kidney–lung interactions and complement activation in AKI through a bibliographic search of PubMed, Scopus, and Web of Science. Most available data derive from experimental studies or intensive care unit (ICU) populations, often reported in reviews. We further report our real-world experience in a non-ICU nephrology setting, including 186 consecutive patients with AKI. Pulmonary involvement was present at hospital admission in 118 patients (63%). AKI stage 1 was observed in 20/118 patients (17%) with pulmonary involvement compared with 18/68 patients (27%) without pulmonary involvement (*p* < 0.001). In conclusion, AKI should be regarded as a systemic disease from its earliest stages. Kidney–lung interactions are clinically relevant even in mild AKI and outside critical care settings, underscoring the need for integrated organ assessment in routine nephrology practice. This review integrates complement activation as a central amplifier of kidney–lung crosstalk and multiorgan dysfunction, bridging experimental evidence with real-world observations from a non-critical care AKI population. By focusing on early AKI stages and the timing of pulmonary involvement, we highlight AKI as an active driver of systemic organ interactions rather than a late consequence of critical illness.

## 1. Introduction

Organ crosstalk describes the dynamic and reciprocal interactions that occur between organs in both physiological and pathological states. Within this framework, the kidney–lung axis represents a particularly important, yet often underappreciated, form of interorgan communication in acute nephrology.

Acute kidney injury (AKI) is not solely a renal disorder but a systemic syndrome capable of inducing remote organ dysfunction, particularly affecting the lung. Pulmonary complications—including acute lung injury (ALI), impaired gas exchange, and increased vascular permeability—commonly accompany AKI and are strongly associated with increased morbidity and mortality, even in patients managed outside the intensive care setting [1,2,3,4,5,6,7].

Physiologically, the kidney and lung cooperate to maintain acid–base homeostasis, regulate vascular tone, ensure adequate oxygen delivery, and modulate neurohormonal pathways including the renin–angiotensin–aldosterone system (RAAS) and sympathetic output [1,4,8,9,10]. During AKI, this finely coordinated regulation is rapidly perturbed, resulting in systemic inflammation, endothelial dysfunction, oxidative stress, and the accumulation of uremic toxins that collectively increase pulmonary vulnerability to injury [1,2,10]. Conversely, primary lung disorders—including acute lung injury, chronic lung disease, hypoxemia, and mechanical ventilation—can precipitate or worsen AKI through hemodynamic compromise, hypoxic renal stress, neurohormonal activation, and systemic cytokine release [11,12,13]. Together, these bidirectional processes play a central role in the development of multiorgan dysfunction during critical illness [13,14].

The heart plays a pivotal intermediary role within this axis. Cardiac congestion, right ventricular dysfunction, and neurohormonal activation amplify the harmful feedback loops between kidney and lung, contributing to fluid overload, venous hypertension, and impaired perfusion [11,15,16,17,18]. Emerging evidence further underscores the contribution of the gut–lung–kidney axis, whereby dysbiosis, disruption of gut barrier integrity, and the release of microbial metabolites influence systemic immune responses and may aggravate both renal and pulmonary injury [19,20,21].

Among the immune and molecular pathways involved in kidney–lung crosstalk, the complement system has increasingly been recognized as a key amplifier of tissue injury.

Complement activation during ischemic, septic, or immune-mediated AKI generates anaphylatoxins (C3a, C5a) and membrane attack complexes that propagate inflammation, promote endothelial and epithelial injury, and recruit neutrophils to remote organs—including the lung. Complement deposition and signalling have been detected in pulmonary tissue after AKI [1,22,23,24,25,26,27] and experimental inhibition of complement pathways attenuates both renal and lung injury, supporting a mechanistic link with potential therapeutic implications [28,29,30,31,32,33,34]. Intracellular complement (“the complosome”) [33,35,36] adds an additional layer of complexity, integrating metabolic stress and immune activation within renal and pulmonary cells [28,29,31,32].

Despite increasing recognition of the kidney–lung axis as an important contributor to multiorgan dysfunction, comprehensive mechanistic understanding and clinical integration remain incomplete.

There is a pressing need to consolidate emerging evidence on immune-molecular mediators—particularly complement activation—that bridge renal and pulmonary injury. Improved comprehension of these complex crosstalk pathways is essential for advancing biomarker discovery, refining risk stratification, and developing targeted therapies to mitigate acute organ injury and improve outcomes beyond the intensive care unit.

To address this gap, a comprehensive bibliographic search was conducted across major databases including PubMed, Scopus, and Web of Science, focusing on recent experimental and clinical studies investigating kidney–lung interactions in acute kidney injury and acute lung injury contexts. Search terms combined keywords related to “kidney–lung crosstalk,” “acute kidney injury,” “acute lung injury,” “complement system,” “inflammation,” and “multiorgan dysfunction.” Priority was given to articles published within the past decade, along with seminal classic papers providing foundational insights. The gathered literature was critically appraised to synthesize current understanding of physiological interactions, molecular mechanisms, and experimental models, with particular emphasis on the translational relevance of complement pathways in kidney–lung injury. The majority of retrieved papers were largely derived from intensive care populations and experimental models, often reported in reviews.

This review synthesizes the physiological basis, mechanistic pathways, experimental models, biomarkers, and therapeutic implications of kidney–lung crosstalk, with a particular emphasis on complement activation as a translationally relevant mediator of multiorgan dysfunction. Unlike previous reviews, which largely focused on ICU populations or isolated mechanisms, this work integrates complement activation into a unified framework of kidney–lung crosstalk and combines mechanistic insights with real-world data from a non-ICU nephrology cohort, emphasizing early AKI stages and the temporal dynamics between renal and pulmonary involvement.

This review extends beyond existing literature by integrating complement activation into a unified framework of kidney–lung crosstalk and multiorgan dysfunction, while also incorporating real-world clinical observations from a non–critical care nephrology setting. In contrast to prior reviews predominantly focused on ICU populations or isolated mechanistic pathways, we emphasize early stages of AKI and the temporal relationship between renal and pulmonary involvement.

## 2. Kidney–Lung Crosstalk and Multiorgan Dysfunction: Physiology and Pathophysiology

### 2.1. Physiological Interactions

Under normal conditions, the kidney and lung function as tightly coordinated organs with shared roles in maintaining acid–base homeostasis, vascular tone, oxygen transport, fluid balance, and systemic metabolic stability. Their integration is essential for adapting to physiological stressors and ensuring adequate delivery of oxygen and nutrients to tissues [1,4,37,38].

The lungs regulate the partial pressure of carbon dioxide (pCO_2_) through alveolar ventilation, providing immediate adjustment of systemic pH. The kidneys complement this by modulating plasma bicarbonate through tubular reabsorption and hydrogen ion secretion, enabling slower but more durable pH correction [6,9]. Together, they create a dual-buffered system that stabilizes the internal environment under varying metabolic demands [35].

Fluid balance and vascular tone represent another critical axis of coordination. The pulmonary circulation responds to changes in blood flow and oxygen tension by adjusting vascular resistance, mediated by nitric oxide, prostaglandins, and angiotensin-converting enzyme (ACE) [39]. Simultaneously, the kidneys regulate extracellular volume and systemic vascular resistance through the renin–angiotensin–aldosterone system (RAAS), natriuretic peptides, and sympathetic nervous system activity. These interactions maintain preload, afterload, and cardiac output within physiological ranges [36,39].

Oxygen transport also depends on kidney–lung integration. Adequate pulmonary gas exchange determines arterial oxygen saturation, which is directly sensed by renal peritubular fibroblasts. Through the hypoxia-inducible factor (HIF) pathway, the kidney modulates erythropoietin (EPO) production, aligning hemoglobin synthesis with systemic oxygen demand. Because the renal medulla operates near hypoxic thresholds even under resting conditions, fluctuations in arterial oxygenation strongly influence renal oxygen delivery and susceptibility to injury [40,41,42].

Shared epithelial architecture further underpins the relationship between the kidney and lung. Both alveolar epithelial cells and renal tubular epithelial cells exhibit polarized membranes, tight junctions, and similar distributions of water and solute channels such as aquaporins and ENaC. This structural parallelism partly explains why systemic inflammatory and metabolic insults often affect both organs in similar ways [4,41].

Neurohormonal integration is essential to their coordinated physiology. Activation of RAAS, sympathetic output, or vasopressin secretion affects both renal and pulmonary vascular beds, modulating sodium retention, vascular resistance, bronchial tone, and water homeostasis. These structural features may confer a degree of parallel vulnerability to systemic inflammatory and metabolic stressors, but it is important to acknowledge that the nephron and the alveolus are anatomically and functionally distinct units, and that inflammatory responses are primarily driven by context-dependent immune and microenvironmental factors [4,8,10,14,43].

#### Complement in Physiologic Kidney–Lung Crosstalk

**Figure 1:**
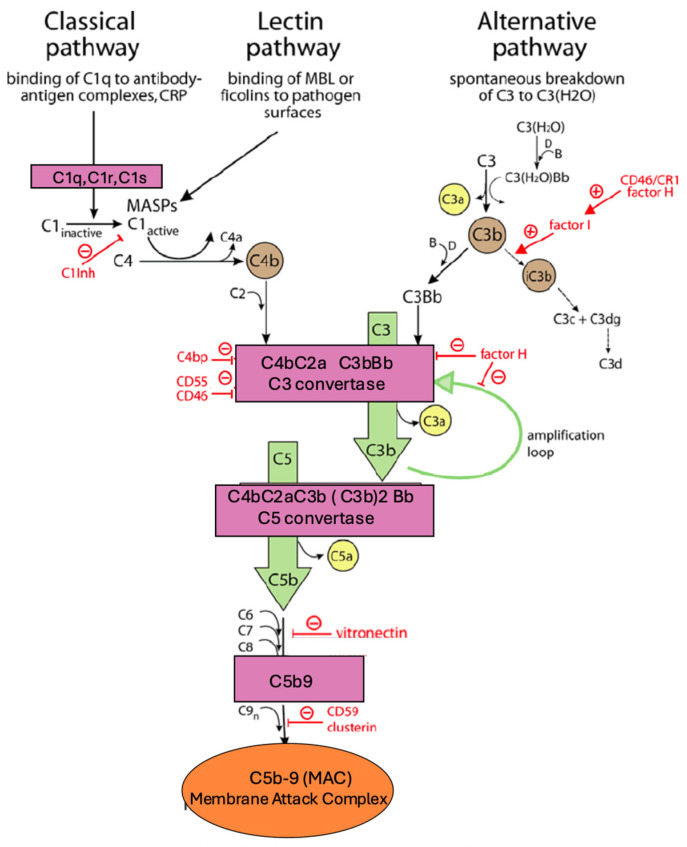
Schematic representation of the complement cascade activation pathways. Overview of complement activation pathways and regulatory mechanisms. Schematic representation of the classical, lectin, and alternative complement pathways, all converging on the formation of the C3 convertase and downstream C5 convertase, ultimately leading to assembly of the membrane attack complex (MAC, C5b–9). Pink boxes indicate complement enzymatic complexes, green arrows represent downstream activation and amplification steps, yellow circles denote anaphylatoxins (C3a, C5a), and the orange oval represents the terminal MAC. The alternative pathway amplification loop is highlighted in green. Red ⊖ symbols and red labels indicate en-dogenous regulatory and inhibitory proteins, including C1 inhibitor (C1inh), CD46, CD55, CD59, complement receptor 1 (CR1), factor H, factor I, vitronectin, and clusterin, which limit excessive complement activation and protect host tissues. Abbreviations: MBL, mannose-binding lectin; MASPs, MBL-associated serine proteases; MAC, membrane attack com-plex. Cellular and temporal dynamics are simplified for clarity.

Although traditionally viewed as a defense mechanism against pathogens, complement participates in physiological homeostasis within the kidney–lung axis. Basal levels of complement components—including C3, C5, factor H, and mannose-binding lectin—are continuously produced by hepatocytes, renal tubular cells, and alveolar macrophages. (Figure 1) These components contribute to immune surveillance, clearance of apoptotic cells, and maintenance of epithelial integrity [28,29,31,34,35,44].

The diagram illustrates the three main complement activation pathways—classical (triggered by C1q binding to antibody–antigen complexes or CRP), lectin (initiated by mannose-binding lectin [MBL] or ficolins binding pathogen surfaces), and alternative (spontaneous C3 hydrolysis to C3H_2_O)—converging on C3 and C5 convertases. Central activation generates anaphylatoxins C3a and C5a, while terminal pathway components (C5b-C9) form the membrane attack complex (MAC, C5b-9). Regulatory proteins (factor H, factor I, CD46, CD55, CD59, C1Inh, C4bp, vitronectin, clusterin) inhibit excessive activation, with amplification loops enhancing C3/C5 cleavage.

In the lungs, low-level complement activity assists with recognition of inhaled particles and supports macrophage function without inducing inflammation. In the kidneys, complement contributes to immune homeostasis along the tubular lumen and interstitium. Importantly, regulatory proteins such as CD46, CD55, and CD59 are highly expressed in both organ systems to prevent uncontrolled activation under resting conditions [44,45,46,47].

This physiological complement tone provides a poised system that can rapidly respond to injury or infection. However, in the setting of metabolic stress, hypoxia, or inflammation, complement can shift from a homeostatic role to a pathogenic one, amplifying kidney–lung crosstalk during AKI and respiratory disease.

### 2.2. Pathophysiological Disruption in AKI

Acute kidney injury (AKI) disrupts the tightly balanced physiological coordination between the kidney and lung, generating systemic disturbances that rapidly extend beyond the renal parenchyma. Even mild or transient AKI can induce widespread metabolic, inflammatory, hemodynamic, and endothelial alterations that predispose the lung to secondary injury. The transition from normal physiology to pathological crosstalk begins early in the course of AKI and often precedes clinically detectable pulmonary symptoms.

Recent mechanistic studies demonstrate that even mild or transient AKI can induce systemic inflammatory and metabolic disturbances, leading to early pulmonary changes before overt clinical symptoms [22,44,48,49].

A landmark study by Komaru et al. [4,50] showed that AKI triggers rapid intravascular neutrophil retention in lung capillaries, causing hypoxemia by reducing capillary blood flow, independent of overt pulmonary edema. This process is mediated by CXCL2 released from activated classical monocytes, and occurs within minutes of AKI onset, preceding clinical respiratory compromise [22,50,51].

Loss of renal clearance leads to the accumulation of uremic toxins, organic acids, and middle-molecule mediators such as indoxyl sulfate, p-cresyl sulfate, mitochondrial DNA, and inflammatory cytokines. These substances circulate systemically and exert adverse effects on pulmonary endothelial cells, impairing barrier integrity and amplifying local inflammation. Renal tubular epithelial injury triggers immediate release of IL-6, IL-1β, TNF-α, chemokines such as CXCL1 and CXCL2, and DAMPs like HMGB1, ATP, and extracellular histones. These mediators activate lung endothelial cells, upregulate adhesion molecules, and promote neutrophil recruitment into alveolar capillaries, setting the stage for microvascular congestion and impaired gas exchange [1,28,46,52].

Oxidative stress is another major hallmark of AKI-related systemic dysfunction. Mitochondrial injury within renal epithelial cells leads to excessive production of reactive oxygen species (ROS), which further destabilize mitochondrial function and propagate systemic oxidative injury. Circulating ROS and mitochondrial DAMPs reach the lung, where they activate pattern-recognition receptors, promote NETosis, a distinct, regulated form of neutrophil cell death characterized by the release of neutrophil extracellular traps (NETs) [48]. NETs are web-like structures composed of decondensed chromatin decorated with antimicrobial proteins such as histones, neutrophil elastase, and myeloperoxidase. This process is triggered by various stimuli, including pathogens, immune complexes, cytokines, and microcrystals, and serves as a mechanism for trapping and neutralizing microbes during the innate immune response [22,46,48,49,51] that disrupt the endothelial glycocalyx. The combined effect is increased vascular permeability and interstitial edema.

Hemodynamic instability contributes significantly to pulmonary vulnerability during AKI. Neurohormonal activation—including RAAS, sympathetic outflow, and vasopressin release—drives vasoconstriction, sodium retention, and fluid overload. These changes increase pulmonary capillary hydrostatic pressure, promoting fluid extravasation even before overt volume overload becomes clinically apparent. In addition, reduced renal oxygen delivery and impaired autoregulation exacerbate systemic metabolic stress, increasing lactate production and worsening tissue hypoxia [1,35,41].

The pulmonary consequences of these processes include alveolar–capillary thickening, neutrophil retention, limited but functionally significant interstitial edema, impaired compliance, and ventilation–perfusion mismatch. Notably, histological studies show that AKI-induced lung injury often occurs without the classic diffuse alveolar damage seen in ARDS; instead, it presents as subtle yet clinically relevant microvascular and endothelial dysfunction [1,35,36,41].

#### Complement Dysregulation During AKI

Complement activation is a central amplifier of the pathological processes initiated by AKI. Under physiological conditions, complement activity is tightly regulated by a group of membrane-bound complement regulatory proteins inhibitors such as CD46, CD55, and CD59 that protect cells from complement-mediated damage. However, renal ischemia, tubular necrosis, uremic toxin accumulation, and systemic inflammation trigger uncontrolled activation of the alternative and lectin pathways [22,44,48,53,54].

During AKI, complement dysregulation manifests through a series of interconnected alterations. Excessive generation of C3a and C5a leads to their systemic dissemination, promoting leukocyte recruitment, endothelial activation, and cytokine release in remote tissues including the kidney and lung. Concurrently, C3 fragments and the membrane attack complex C5b-9 (MAC) deposit on renal tubules and pulmonary microvascular endothelium, contributing to epithelial and endothelial injury.

This occurs against a backdrop of reduced expression and functional exhaustion of complement regulatory proteins, rendering both organs more vulnerable to complement-mediated damage. The C5a–C5aR1 axis plays a central role by driving neutrophil retention and chemotaxis within alveolar capillaries while amplifying pro-inflammatory signalling in lung macrophages [1]. Complement–DAMP synergy further exacerbates this process, as mitochondrial DNA, HMGB1, and extracellular histones released during AKI enhance complement activation—particularly via the lectin pathway—and increase the efficiency of C3 and C5 cleavage, thereby fuelling a vicious cycle of inflammation.

This dysregulated complement activity is detectable early in AKI—even before creatinine rises—and correlates with the onset of pulmonary endothelial dysfunction. Experimental models demonstrate that complement inhibition, particularly targeting C3 or C5aR1, reduces pulmonary leukocyte sequestration, improves microvascular perfusion, and attenuates interstitial edema. Thus, complement dysregulation is not merely an associated feature of AKI but a mechanistic driver of kidney–lung crosstalk and an attractive therapeutic target [22,49,51,52,53,54].

### 2.3. Bidirectional Dysfunction

Kidney–lung interactions are inherently bidirectional, with injury in either organ capable of precipitating dysfunction in the other. This interconnected vulnerability reflects shared hemodynamic pathways, overlapping immune responses, metabolic dependencies, and neurohormonal regulation. Whether injury originates in the kidney or in the lung, disruptions propagate rapidly across organ systems and can culminate in multiorgan dysfunction.

#### 2.3.1. AKI → Lung Dysfunction (Forward Crosstalk)

Acute kidney injury often precedes or aggravates pulmonary complications through several converging mechanisms. Tubular epithelial injury triggers systemic inflammation [50,53] characterized by release of IL-6, IL-1β, TNF-α, CXCL1, CXCL2, HMGB1, and mitochondrial DNA, which act as chemoattractants, primarily recruiting neutrophils and other immune cells to sites of infection or injury [22,55]. HMGB1 is a multifunctional nuclear protein that acts as an inflammatory mediator when it is released from cells, either passively through cell death (Netosis) or actively through secretion from stressed or activated immune cells, where it acts as a damage-associated molecular pattern (DAMP) [48,54,56]. It can recruit immune cells, often in complex with chemokines like CXCL12. Together, these circulating mediators activate pulmonary endothelial cells, upregulate adhesion molecules, and promote neutrophil sequestration in alveolar capillaries. The resulting microvascular congestion impairs gas exchange and contributes to ventilation–perfusion mismatch [52,54].

Metabolic disturbances secondary to AKI, including oxidative stress and mitochondrial dysfunction, further compromise pulmonary endothelial integrity. Neurohormonal activation—particularly RAAS, sympathetic output, and vasopressin release—promotes sodium and water retention, venous congestion, and increases pulmonary hydrostatic pressure [57]. The lung becomes more susceptible to edema, reduced compliance, and impaired oxygenation [22].

Importantly, lung injury during AKI often develops without classic diffuse alveolar damage, presenting instead with subtle endothelial dysfunction, septal thickening, neutrophil retention, and interstitial edema that significantly impact respiratory function even when imaging findings appear minimal [22,48,49].

#### 2.3.2. Lung Injury → AKI (Reverse Crosstalk)

Primary lung disorders, including pneumonia, ARDS, COPD exacerbations, and ventilator-induced lung injury, frequently contribute to AKI. Hypoxemia reduces renal cortical and medullary oxygenation, while hypercapnia induces renal vasoconstriction and reduces filtration [46,50]. These effects impair renal autoregulation and lead to ischemic tubular injury [50,51,53].

Mechanical ventilation introduces unique risks [13,58,59,60]. High airway pressures and alveolar overdistension increase intrathoracic pressure, reduce venous return, and lower renal perfusion. “Biotrauma” generated during ventilator-induced lung injury results in systemic cytokine release that directly injures renal endothelial and tubular cells [59,60,61]. Patients with ALI or ARDS often develop AKI not solely due to hemodynamic compromise but also because of systemic inflammatory cascades originating in the lung.

Metabolic consequences of lung injury, such as increased lactate production and mitochondrial injury, similarly impact renal cellular energetics and exacerbate susceptibility to AKI [22,50,51,53].

#### 2.3.3. The Heart as an Intermediary Node

The heart serves as a major integrator within kidney–lung crosstalk. Right ventricular dysfunction due to pulmonary hypertension elevates central venous pressure, directly impairing renal perfusion and filtration [15,16,17,18]. Left ventricular dysfunction increases pulmonary venous pressure, promoting pulmonary congestion and worsening gas exchange [57]. The combined hemodynamic burden perpetuates a cycle of volume overload, reduced perfusion, and multiorgan dysfunction [19,20,62].

Neurohormonal responses to cardiac dysfunction—RAAS activation, sympathetic surge, and vasopressin release—exacerbate both renal sodium retention and pulmonary edema, creating a self-amplifying cardiorenal-pulmonary syndrome [20,62].

#### 2.3.4. Gut–Lung–Kidney Axis

The gastrointestinal tract provides an additional layer of interconnected regulation within multiorgan crosstalk during AKI.

Experimental and clinical evidence indicate that AKI disrupts gut barrier function and alters the microbiome, promoting translocation of microbial products such as lipopolysaccharides and increasing systemic inflammation [59]. Dysbiosis increases production of gut-derived uremic toxins (e.g., indoxyl sulfate), which impair both renal tubular function and pulmonary endothelial integrity [20,58], possible link of early gut-lung-kidney axis.

In addition to their direct toxic effects, gut-derived metabolites and microbial products may act as upstream triggers of innate immune activation, including complement and pattern-recognition receptor pathways, thereby amplifying inflammatory signaling across organs.

These processes may act in concert with innate immune activation, including complement pathways, thereby amplifying systemic inflammatory signaling across organs.

Alterations in gut barrier integrity, microbiome composition, and gut-derived uremic toxin production may interact with complement activation and cytokine signaling, thereby amplifying inflammatory responses across renal and pulmonary compartments. In the setting of AKI, this gut–lung–kidney axis may contribute to systemic organ crosstalk by linking immune, metabolic, and endothelial pathways.

In this phase, the liver, as a central organ in metabolism and immune regulation, can modulate the systemic inflammatory response and the clearance of circulating toxins and cytokines, thereby influencing the severity and progression of both kidney and lung injury [63].

Through its role in complement protein synthesis and detoxification processes, hepatic involvement may further modulate the intensity and persistence of kidney–lung inflammatory interactions.

Conversely, lung inflammation alters intestinal perfusion, increases permeability, and drives microbial translocation, which then aggravates renal inflammation. Microbiome-mediated immune modulation—including changes in T cell phenotypes and cytokine profiles—further links renal and pulmonary susceptibility to injury [20,58]. Although still incompletely characterized in acute nephrologic settings, this axis highlights the systemic nature of kidney–lung interactions beyond direct organ-to-organ signaling.

**Figure 2:**
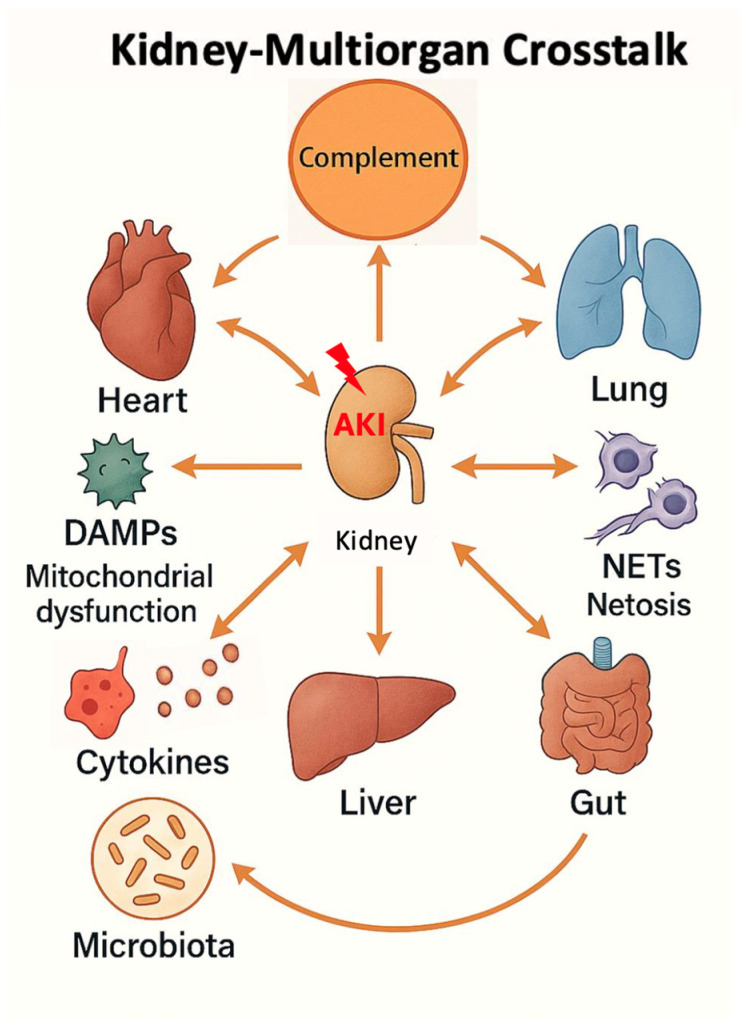
Overview of kidney–lung crosstalk within the multiorgan network.

Although mechanistic and clinical data remain limited, this bidirectional gut–lung–kidney interaction supports a systemic view of AKI as a driver of multiorgan immune dysregulation rather than a purely renal condition (Figure 2).

#### 2.3.5. Complement in Forward and Reverse Crosstalk

Complement activation is a central mechanism that amplifies bidirectional kidney–lung dysfunction. In AKI, activation of the alternative and lectin pathways generates high systemic levels of C3a and C5a, which travel to the lung and initiate endothelial activation, permeability changes, and neutrophil anchoring within alveolar capillaries. These same mediators depress pulmonary microcirculatory flow and enhance cytokine release, aggravating early lung injury [11,13,14].

In the reverse direction, lung injury activates local complement within the alveolar space. Alveolar macrophages and pulmonary epithelial cells produce C3 and factor B in response to inflammatory stimuli, and C5a generated locally spills into the systemic circulation. This spillover enhances renal endothelial activation, tubular chemokine expression, and leukocyte recruitment, setting the stage for AKI [1,26,27].

Complement further integrates into the cardiorenal–pulmonary network, acting as a common inflammatory and vascular mediator across organs. In the setting of pulmonary hypertension or right ventricular dysfunction, complement activation contributes to vascular remodelling and endothelial injury, thereby worsening pulmonary vascular load and impairing right heart function [64,65]. Along the gut–lung–kidney axis, microbial translocation and dysbiosis enhance complement pathway activity, particularly via mannose-binding lectin and the lectin pathway, amplifying systemic inflammation and organ vulnerability (Figure 2).

AKI acts as the central hub propagating injury signals to lung and heart through complement activation, DAMPs, cytokines, mitochondrial dysfunction, NETosis, and neutrophil extracellular traps (NETs). Bidirectional arrows depict kidney-to-lung forward crosstalk (via circulating mediators causing pulmonary endothelial injury and microvascular congestion) and lung-to-kidney reverse crosstalk. Additional nodes represent gut microbiota dysbiosis and liver involvement amplifying systemic inflammation, highlighting the integrated cardiorenal–pulmonary–gut axis in multiorgan dysfunction during AKI. Complement is emphasized as the principal mechanistic bridge linking renal injury to remote pulmonary and cardiac complications.

DAMPs released from any injured organ within this network, including the kidney, lung, heart, or gut, further potentiate complement cleavage and activation. This complement–DAMP interplay establishes a self-amplifying inflammatory loop that propagates damage across the cardiorenal–pulmonary continuum and favours progression to multiorgan dysfunction (Figure 2) [24,26,29,30].

This bidirectional amplification [13,14] makes complement a mechanistic bridge [33,35] between kidney and lung injury and positions complement inhibitors as promising therapeutic strategies for multiorgan dysfunction [32,33,46].

## 3. Integrated Mechanistic Role of Complement in Kidney–Lung Crosstalk

**Figure 3:**
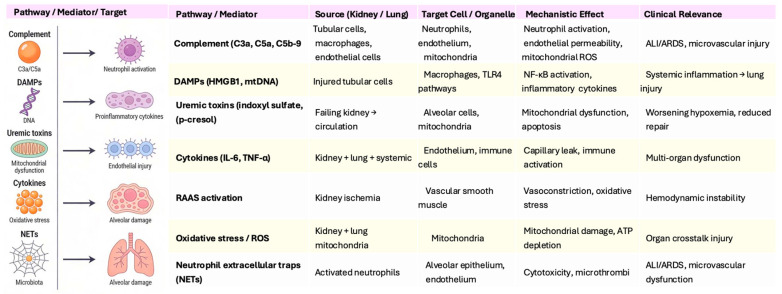
Molecular pathways and cellular effects in kidney–multiorgan crosstalk. Abbreviations: ALI, Acute Lung Injury; ARDS, Acute Respiratory Distress Syndrome; ATP, Adenosine Triphosphate; DAMPs, Damage-Associated Molecular Patterns; HMGB1, High-Mobility Group Box 1; IL-6, Interleukin-6; mtDNA, Mitochondrial DNA; NF-κB: Nuclear Factor Kappa B; NETs: Neutrophil Extracellular Traps; RAAS: Renin–Angiotensin–Aldosterone System; ROS: Reactive Oxygen Species; TNF-α: Tumor Necrosis Factor Alpha.

Complement activation acts as a central amplifier of kidney–lung crosstalk, integrating inflammatory, endothelial, metabolic, and immune pathways that link injury in one organ to dysfunction in the other. The mechanisms involve uncontrolled generation of anaphylatoxins, impaired regulation of complement, synergy with DAMPs and cytokines, and downstream endothelial and epithelial injury. These highlights complement not just as a feature of either kidney or lung injury, but as the core driver of bidirectional multiorgan dysfunction (Figure 3).

Schematic overview of the main molecular and cellular pathways implicated in kidney–lung crosstalk during acute kidney injury (AKI). Renal tubular injury promotes the release of inflammatory mediators, including complement components, damage-associated molecular patterns (DAMPs), uremic toxins, and cytokines, which act on immune cells, endothelium, and mitochondria, contributing to pulmonary endothelial and epithelial injury. Activation of the renin–angiotensin–aldosterone system (RAAS), oxidative stress, mitochondrial dysfunction, and neutrophil extracellular trap (NET) formation further amplify systemic inflammation and microvascular damage. Together, these pathways provide a mechanistic framework linking early renal injury to pulmonary involvement and multi-organ dysfunction.

Key mediators released during kidney-lung crosstalk: complement anaphylatoxins, DAMPs, uremic toxins, proinflammatory cytokines, oxidative stress, and NETs. Complement-driven neutrophil activation, DAMP-induced cytokine production, mitochondrial dysfunction, and NETosis collectively promote endothelial injury and alveolar damage, while gut microbiota alterations further amplify systemic inflammation.

### 3.1. Complement-Mediated Mechanisms in AKI–Lung Dysfunction

AKI triggers complement activation primarily through the alternative and lectin pathways, producing circulating C3a, C5a, and C5b-9 fragments [32,52,54]. These reach the lung where C5a binds to C5aR1 on pulmonary endothelial cells, alveolar macrophages, and neutrophils, inducing endothelial activation, cytokine release, and adhesion molecule expression [49,52,54]. This promotes neutrophil retention enhanced by CXCL2 signaling, causing microvascular congestion and impaired oxygen exchange. C5b-9 disrupts endothelial membranes, increasing permeability and inducing interstitial edema [49]. Kidney-derived DAMPs such as mitochondrial DNA and HMGB1 amplify complement activation and pulmonary inflammation via TLR4 and NF-κB, establishing a positive feedback loop that worsens lung injury, even in subclinical kidney damage [52,54]. Complement inhibition reduces neutrophilia, restores endothelial integrity, and improves lung function, underscoring its mechanistic bridging role [66,67].

### 3.2. Complement Activation in Lung-Originated Renal Injury

Pulmonary infections and inflammatory lung injury stimulate local complement production by alveolar macrophages, pneumocytes, and infiltrating immune cells. Complement fragments, especially C5a, enter systemic circulation and act on renal endothelial and tubular epithelial cells by binding C5aR1, activating NF-κB, inducing cytokine release, leukocyte recruitment, tubular apoptosis, and microvascular congestion [59]. The terminal complement complex C5b-9 deposits in renal tissue causing sublytic injury, cytoskeletal disruption, and increased permeability that exacerbate proteinuria and maladaptive repair. Lung-derived DAMPs (mitochondrial DNA, HMGB1, histones) potentiate complement activation and amplify TLR signaling in the kidney in a feedback loop, accelerating inflammation and injury [22,23,25,59].

Mechanical ventilation enhances complement activity, with ventilator-induced complement deposition driving renal injury. Blockade of complement reduces renal inflammation and improves perfusion, indicating complement as a key mechanistic link in lung-to-kidney crosstalk [25,26,60].

### 3.3. Complement as an Early Systemic Signal

Complement activation is rapidly triggered during renal ischemia, tubular necrosis, pulmonary infection, or inflammatory lung injury [25,68]. The alternative and lectin pathways produce high levels of C3a and C5a, which circulate systemically to pulmonary and renal microvasculature. These anaphylatoxins are potent chemoattractants and immune modulators, priming neutrophils, monocytes, and macrophages, thus propagating inflammation before clinical organ dysfunction emerges [28,30,31,32].

### 3.4. The C5a–C5ar1 Axis as a Bidirectional Amplifier

The C5a–C5aR1 signalling pathway is the principal effector connecting kidney and lung injury. C5a binds C5aR1 on endothelial cells, alveolar macrophages, neutrophils, and renal tubular epithelial cells, activating NF-κB–dependent transcription to increase cytokine production (IL-6, TNF-α, IL-1β), enhance endothelial adhesion molecules, promote neutrophil retention in capillaries, and raise vascular permeability and edema [22,54].

This establishes a positive feedback loop where complement activation accelerates injury in both organs.

### 3.5. Complement–Damp Synergy

Complement strongly interacts with DAMPs such as HMGB1, extracellular ATP, mitochondrial DNA, and histones, all released during AKI and lung injury. DAMPs activate TLRs on endothelial and immune cells, accelerating complement activation via the lectin pathway. Meanwhile, C3a and C5a amplify TLR signaling, and NF-κB activation elevates local complement production, creating a mutually reinforcing cycle that drives rapid and progressive kidney–lung injury [25,26,29,32].

### 3.6. Endothelial Vulnerability to Complement Injury

Endothelial cells in kidney and lung are highly susceptible to complement-mediated damage. Sublytic C5b-9 deposition disrupts cytoskeletal architecture, elevates intracellular calcium, and impairs tight junctions, leading to microvascular leakage, interstitial edema, impaired oxygen diffusion, microthrombi formation, and reduced capillary perfusion—characteristic microvascular dysfunction of kidney–lung crosstalk [2,69,70].

### 3.7. The Complosome: Intracellular Complement Activation

An intracellular complement system, the complosome, exists in renal tubular cells, alveolar macrophages, and endothelial cells [71]. Intracellular cleavage of C3 and C5 drives metabolic reprogramming via mTORC1 activation, cytokine production, stress responses, and persistent inflammation, explaining ongoing organ injury and immune activation after systemic complement activation subsides [27,71,72,73].

### 3.8. Complement as the Central Link to Multiorgan Dysfunction

Complement activation provides a unifying mechanistic framework explaining how AKI causes early lung microvascular injury, how lung injury triggers rapid kidney decline, why dual insults cause severe disease, and why systemic inflammation frequently leads to kidney–lung multiorgan dysfunction [35,74]. Because of its central upstream role, complement is a promising therapeutic target in this complex interplay.

## 4. Experimental Animal Models of Kidney–Lung Crosstalk

Experimental models provide essential mechanistic insights into how kidney and lung injury develop, interact, and amplify one another. They reproduce key clinical conditions—ischemia, uremia, sepsis, lung injury, mechanical ventilation—and demonstrate that injury in one organ is sufficient to provoke dysfunction in the other. These models establish causal relationships, reveal specific molecular pathways, and highlight complement as a pivotal amplifier of cross-organ injury.

### 4.1. Ischemia–Reperfusion Injury (IRI)

Renal ischemia–reperfusion injury is the most widely studied model of AKI and a central platform for understanding forward kidney–lung crosstalk. Transient clamping of the renal pedicle in murine induces tubular hypoxia, endothelial dysfunction, and rapid release of DAMPs during reperfusion [66,75,76].

These signals initiate a systemic inflammatory cascade that affects the lung within hours.

Key pathophysiologic features of IRI can be described as a coordinated cascade rather than isolated events. During reperfusion, DAMPs such as HMGB1, mitochondrial DNA, ATP, and extracellular histones are released into the circulation and activate TLR4 on pulmonary endothelial and immune cells, thereby priming the lung for injury [77]. At the same time, CXCL2 produced by infiltrating monocytes promotes pulmonary neutrophil retention and the formation of “neutrophil trains” within alveolar capillaries, which impairs microvascular perfusion and gas exchange. The pulmonary endothelial barrier is rapidly compromised, with increased permeability, glycocalyx degradation, and interstitial edema occurring early, even in the absence of classical alveolar flooding on imaging [50].

In parallel, IRI induces widespread mitochondrial dysfunction and oxidative stress in both kidney and lung tissues, leading to metabolic failure and reduced cellular resilience. This is further amplified by a renal-driven cytokine response, as IL-6, TNF-α, and IL-1β released from the injured kidney accelerate pulmonary inflammation and promote additional recruitment of immune cells into the lung [75,78].

This model demonstrates that AKI alone—without lung insult—can rapidly precipitate microvascular and inflammatory changes in the lung.

#### Complement Activation in IRI Models

Complement activation is an early and central feature of the IRI model [79,80].

Renal ischemia triggers robust activation of the alternative and lectin pathways, producing high levels of C3a and C5a within minutes of reperfusion and the membrane attack complex (MAC, C5b-9). These mediators amplify sterile inflammation by promoting immune cell recruitment, cytokine release, oxidative stress, and endothelial and parenchymal cell injury [66,75,81].

Experimental studies across organ systems—including liver, kidney, heart, and brain—demonstrate that complement activation is an early and necessary event in IRI. Inhibition or genetic deficiency of complement components (e.g., C3, C5, factor B, C3aR, C5aR, or MAC) consistently reduces tissue injury, leukocyte infiltration, and subsequent fibrosis in animal models [4]. The alternative pathway, in particular, acts as an amplifier of complement activation and tissue damage, while the terminal MAC is directly cytotoxic to parenchymal cells in organs such as the kidney and liver [66,75,81].

These mediators enhance CXCL2-driven neutrophil retention in pulmonary capillaries, increase pulmonary endothelial permeability, promote cytokine release from alveolar macrophages, and contribute to C5b-9 deposition in lung microvasculature.

C5a–C5aR1 signaling is particularly important in IRI-induced lung injury. Blocking C5aR1 reduces pulmonary neutrophilia, improves gas exchange, and decreases endothelial damage. Mice lacking C3 or treated with complement inhibitors show attenuated lung injury after IRI, underscoring complement’s mechanistic role [25,26,82].

### 4.2. Uremic and Bilateral Nephrectomy Models

Uremic animal models—including bilateral nephrectomy and adenine-induced renal failure—reproduce chronic kidney dysfunction and toxin accumulation independent of ischemic injury. These models demonstrate how systemic uremic milieu alone can trigger pulmonary dysfunction [25,79,82,83,84].

Bilateral nephrectomy models have been used to isolate the effects of absent renal clearance without confounding ischemic injury. Hoke et al. showed that bilateral nephrectomy in mice leads to acute renal failure with marked increases in circulating proinflammatory cytokines (e.g., IL-6, IL-1β), which directly mediate pulmonary injury characterized by vascular congestion and neutrophil infiltration [85]. Importantly, administration of anti-inflammatory cytokine IL-10 ameliorated lung injury, confirming that the systemic uremic milieu alone, via cytokine imbalance, can trigger pulmonary dysfunction independent of ischemic injury [80,85,86]. Doi et al. further elucidated the mechanism, demonstrating that the HMGB1-TLR4 pathway is activated after bilateral nephrectomy, leading to neutrophil infiltration and increased vascular permeability in the lung [86]. Blockade of HMGB1 or TLR4 reduced pulmonary injury, again supporting the direct role of uremic toxin-driven systemic inflammation in lung pathology [87].

Major mechanisms in uremic lung injury reflect a diffuse, systemic inflammatory and metabolic burden. The accumulation of uremic toxins such as indoxyl sulfate and p-cresyl sulfate drives pulmonary endothelial activation and oxidative stress, setting the stage for structural and functional damage [79,83,86]. In parallel, pulmonary neutrophilia and macrophage activation mirror the underlying systemic inflammatory state and further amplify local injury [88].

These inflammatory changes are accompanied by thickening of the alveolar–capillary interface, resulting from endothelial injury and expansion of the interstitial matrix, which impairs gas exchange [89]. Fluid handling is also affected: sodium retention and impaired lymphatic drainage contribute to increased lung water content and a tendency toward interstitial edema [58,89]. Finally, immune cells, particularly neutrophils and monocytes, become primed and exhibit heightened responsiveness to inflammatory stimuli, making the lung more vulnerable to subsequent hits.

These findings demonstrate that even in the absence of ischemia, kidney failure induces a systemic pro-inflammatory state that directly affects lung integrity.

#### Complement Activation in Uremic Models

In uremic bilaterally nephrectomised animal models, the role of complement is fundamentally altered due to the absence of renal tissue, which is a significant site of both local complement synthesis and activation [85]. After bilateral nephrectomy, systemic complement proteins (primarily produced by the liver) remain present, but the kidney’s contribution to local complement production and regulation is lost [63]. This results in a shift in complement activity from local renal effects to systemic consequences, particularly in the context of uremia [82,88,90].

Experimental studies demonstrate that complement activation in uremic states contributes to systemic inflammation, vascular dysfunction, and organ injury outside the kidney. In nephrectomised models, complement components such as C3a and C5a, as well as the membrane attack complex (MAC), are implicated in promoting proinflammatory and profibrotic responses, exacerbating the uremic milieu and contributing to complications such as cardiovascular remodeling and immune dysregulation [73,85,88,91]. The absence of renal clearance and local complement regulation further amplifies these effects.

Importantly, animal studies using complement-deficient or complement-inhibited models show that reduced complement activation mitigates systemic inflammation and fibrosis in uremic conditions, supporting the pathogenic role of complement in extra-renal complications of uraemia [12,67,73,79,92].

Complement activity is markedly increased in uremic states due to impaired clearance. High circulating levels of C3a, C5a, and sC5b-9 reflect persistent complement activation. Complement contributes to lung injury in uremia by enhancing endothelial activation and permeability, promoting neutrophil adhesion and migration, increasing oxidative stress in pulmonary tissue, amplifying cytokine and chemokine production, and facilitating interstitial edema through C5b-9–induced microvascular injury.

In adenine-AKI models, experimental data demonstrate that adenine administration in mice leads to increased expression of genes related to both the complement and coagulation cascades, with marked deposition of C3 in fibrotic kidneys, uremia increases complement deposition in the lung, and complement inhibition reduces neutrophil infiltration and septal thickening. This supports complement as a driver of lung injury in chronic kidney dysfunction [27,73,86].

Moreover, pharmacologic inhibition of complement activation, for example with nicotinamide, attenuates these pathways and reduces renal fibrosis, supporting the mechanistic involvement of complement in the disease process [30,32,67,93].

### 4.3. Two-Hit and Synergistic Injury Models

Two-hit models reflect clinical scenarios where AKI or lung injury does not occur in isolation. Instead, an initial insult “primes” the system, making the second insult far more damaging than either alone [8,11,44,91].

These models capture the complex interactions seen in sepsis, postoperative AKI, pneumonia, trauma, and ventilator-associated injury.

When AKI precedes a septic insult, the result is an exaggerated cytokine response, greater pulmonary vascular permeability, and impaired bacterial clearance, reflecting the heightened vulnerability of the lung in the context of prior renal injury [49,94,95]. Similarly, when AKI is followed by mechanical ventilation, there is a markedly enhanced inflammatory response with increased neutrophil retention and worsening gas exchange, consistent with data showing that injurious ventilation magnifies kidney–lung crosstalk and promotes renal apoptosis and microvascular dysfunction [60,96].

The sequence can also be reversed: primary lung injury followed by AKI is associated with intensified renal inflammation, oxidative stress, and tubular damage, underscoring the bidirectional nature of this interaction. Ventilator-induced lung injury (VILI) further synergizes with systemic insults by amplifying circulating cytokines and driving renal microvascular leakage, thereby increasing the risk of ventilation-induced kidney injury. In combined sepsis and AKI models, both kidney and lung exhibit profound metabolic and microvascular dysfunction with multiorgan involvement, mirroring the high morbidity and mortality seen in septic patients with concurrent AKI and respiratory failure [49,97].

These models show that once systemic inflammation is established, both organs act as amplifiers rather than passive recipients of injury. Complement activation is disproportionately intensified in two-hit scenarios, where sequential insults create a synergistic amplification of organ injury [98].

This manifests through accelerated cleavage of C3 and C5, leading to markedly elevated systemic levels of C3a and C5a that propagate widespread inflammation [44,99]. Massive C5a–C5aR1 signaling further drives enhanced cytokine release and neutrophil sequestration in the lung, while increased deposition of C5b-9 in both kidney and lung microvasculature exacerbates endothelial barrier dysfunction and microvascular injury [99,100,101].

Synergy with DAMPs—particularly HMGB1 and mitochondrial DNA—accelerates lectin pathway activation, fueling complement overdrive in an already primed inflammatory environment. Immune cells become hyperresponsive, with neutrophils exhibiting increased NET formation, reduced deformability, and heightened sequestration, compounded by complement overactivation due to diminished regulatory capacity, including reduced factor H activity and CD55 expression. These interconnected mechanisms explain the rapid progression to severe multiorgan dysfunction in sequential injury models [99,100,101].

Complement inhibition between hits significantly attenuates pulmonary edema, nephron injury, and systemic inflammation, confirming complement’s central role as an amplifier of synergistic kidney–lung dysfunction.

## 5. Personal Experience

Introduction

Kidney–lung interactions have been extensively described in critically ill patients and in intensive care unit (ICU) settings, but data from non–critical care nephrology populations remain limited. In everyday clinical practice, AKI is frequently managed in standard nephrology wards, where early organ crosstalk may be overlooked.

In this context, we report our real-world experience.

Methods

Over a 12-month retrospective observational period, 695 patients admitted to the University Hospital Policlinico Tor Vergata (PTV) were evaluated for acute kidney injury (AKI) outside intensive care or high-dependency settings, aiming to assess concomitant pulmonary involvement. Patients were included if AKI was diagnosed and classified per KDIGO criteria, excluding those requiring dialysis, with hemodynamic instability (arterial pressure < 90/60 mmHg), sepsis, unconfirmed AKI, or incomplete data.

Pulmonary involvement was assessed at hospital admission using routinely available clinical and instrumental data. Patients were classified as having pulmonary involvement in the presence of clinical, radiological, and/or gas exchange abnormalities suggestive of lung involvement. Specifically, pulmonary involvement was defined by at least one of the following findings: (i) respiratory symptoms or signs (including dyspnea, tachypnea, or reduced peripheral oxygen saturation); (ii) abnormalities on chest imaging performed for clinical indications (such as interstitial or alveolar opacities or pulmonary congestion); and/or (iii) alterations in arterial or capillary blood gas analysis indicating impaired gas exchange.

Laboratory markers of systemic inflammation, including white blood cell count, C-reactive protein, and procalcitonin were collected at admission and analyzed to characterize inflammatory profiles according to pulmonary involvement status.

Available clinical data were retrieved from electronic charts. Patients expressed consensus for anonymous treatment of data.

Results

A total of 186 patients with acute kidney injury (AKI) were included in the analysis, of whom 118 (63%) exhibited pulmonary involvement at hospital admission (Table 1). Patients with pulmonary involvement were significantly older and more frequently had pre-existing chronic kidney disease compared with those without pulmonary involvement. Inflammatory markers were markedly higher in the pulmonary involvement group, including white blood cell count, C-reactive protein, and procalcitonin (all *p* < 0.001) Pre-existing CKD was also more frequent in the pulmonary involvement group (65% vs. 37%, *p* < 0.05) (Table 1).

Pulmonary involvement was observed across all AKI stages. Notably, 20 patients (17%) with pulmonary involvement presented with AKI stage 1, indicating that lung involvement was already detectable at the earliest stage of kidney injury. Patients with more severe AKI demonstrated significantly higher rates of pulmonary involvement. (63% vs. 26%) (Table 1).

Discussion

The main finding of the present study is that pulmonary involvement is already detectable at the earliest stage of acute kidney injury. When considering the overall AKI stage 1 population, more than half of patients (51.3%) exhibited pulmonary involvement at hospital admission, despite being managed in a non–critical care nephrology setting. This observation challenges the traditional view that lung injury in AKI is primarily a consequence of advanced renal dysfunction or critical illness and instead supports the concept of an early and bidirectional kidney–lung crosstalk.

The presence of pulmonary involvement in mild AKI underscores the role of systemic inflammatory and microvascular mechanisms that may be activated shortly after renal injury. Even limited reductions in renal function may trigger inflammatory mediator release, endothelial dysfunction, and oxidative stress, ultimately affecting distant organs such as the lung.

Importantly, pulmonary involvement was detectable at hospital admission in the very early phase of AKI, even in patients with mild renal dysfunction managed outside critical care settings, underscoring the early onset of kidney–lung interactions.

Together, these findings support a conceptual shift in which AKI is viewed not merely as a target of systemic illness but as an active hub capable of initiating and amplifying multiorgan dysfunction. By integrating complement-mediated mechanisms with clinical observations from a non–critical care cohort, our data suggest that kidney–lung crosstalk begins early in the course of AKI and is not confined to advanced stages or intensive care settings. This perspective helps reconcile experimental evidence with real-world clinical trajectories.

Our findings extend previous observations largely derived from intensive care populations and experimental models, demonstrating that kidney–lung interactions are clinically relevant even in real-world nephrology ward patients.

In this early phase, systemic factors beyond direct kidney–lung interactions may contribute to pulmonary involvement at presentation. Alterations in gut barrier function and microbiome composition occurring soon after AKI onset have been shown to promote systemic inflammation and accumulation of gut-derived uremic toxins, which can affect both renal and pulmonary vascular and epithelial compartments [20,21,63]. Although we did not directly assess intestinal involvement, these mechanisms may partially explain the early pulmonary alterations observed in our non–critical care AKI population.

Clinical Implications and Impact on AKI Management

Although the present study does not aim to modify existing guidelines for the management of acute kidney injury (AKI), it provides clinically relevant insights that may influence everyday nephrology practice. The demonstration of early pulmonary involvement in more than half of patients with AKI stage 1 highlights that lung complications are not restricted to advanced AKI or critical illness but may already be present in mild forms of renal injury managed in non–intensive care settings.

Current AKI classifications, including KDIGO staging, primarily focus on renal function and do not provide guidance regarding the assessment of extra-renal organ involvement, particularly at early stages. Our findings suggest that the kidney–lung interaction should be actively considered even in patients with mild AKI, prompting increased clinical awareness and a more integrated organ-oriented evaluation.

## 6. Conclusions

Kidney–lung crosstalk represents a dynamic, bidirectional network in which injury to one organ rapidly propagates dysfunction in the other through intertwined inflammatory, endothelial, metabolic, hemodynamic, and neurohormonal pathways. Even mild or transient AKI can trigger early pulmonary alterations—neutrophil sequestration, endothelial activation, oxidative stress, and microvascular perfusion defects—that may precede clinically overt respiratory compromise [1,3,4,6].

Likewise, primary lung injuries such as pneumonia, ARDS, or ventilator-induced lung injury can precipitate or worsen AKI through systemic inflammation, hypoxemia, hypercapnia, and mechanical or metabolic stress [8,11,12,13].

Across these diverse pathogenic mechanisms, complement activation functions as an integrative amplifier that links renal injury to downstream multiorgan damage, integrating signals from ischemia, infection, mechanical ventilation, and metabolic injury. Through the generation of C3a, C5a, and C5b-9 and membrane attack complex formation, complement signaling enhances inflammatory responses, disrupts endothelial integrity, and facilitates leukocyte recruitment, disrupts endothelial and epithelial barriers and synergizes with DAMP–TLR pathways to inflammation. The intracellular complement system, or “complosome,” introduces an additional layer of complexity by sustaining cell-intrinsic inflammatory metabolic stress responses and metabolic dysfunction long after the initial insult [1,2,6,7].

Experimental models confirm that complement activation is both an early event and a mechanistic driver of organ crosstalk. In ischemia–reperfusion injury, complement links renal tubular injury to rapid pulmonary microvascular dysfunction. In uremic models, impaired clearance perpetuates complement activation and pulmonary injury. Two-hit models illustrate how complement amplification transforms moderate insults into severe, multiorgan dysfunction, mirroring clinical scenarios such as post-operative AKI, sepsis, and ARDS with renal involvement [4,5].

Clinically, this interplay manifests as mixed cardiogenic and non-cardiogenic pulmonary edema, impaired gas exchange, increased need for ventilatory support, and elevated mortality [5,8,11,14].

The high proportion of patients with pulmonary involvement at the onset of AKI in our cohort (63.5%) is consistent with the growing recognition of lung–kidney crosstalk in acute illness. Experimental and clinical studies have shown that AKI can promote pulmonary edema, increased pulmonary vascular permeability, and inflammatory lung injury, while primary lung disease can, in turn, exacerbate renal dysfunction [9,11].

The prevalence of pre-existing CKD in our cohort mirrors previous reports in hospitalized AKI populations, where CKD stages 3–4 are frequently observed and recognized as major risk factors for developing AKI. Studies have shown that patients with pre-existing CKD are more susceptible to hemodynamic insults, nephrotoxic exposure, and sepsis, and may exhibit different outcome trajectories than those with previously normal renal function (6). The distribution of CKD stages in our series (with a substantial burden of stage 3A–3B and 4) is therefore in line with these data and underscores the vulnerability of this subgroup [3,5,8,14].

Our observation that inflammatory markers (WBC, CRP, and PCT) increase with higher AKI stages is also supported by prior work. Procalcitonin and CRP have been associated with both the development and severity of AKI in septic and postoperative populations, and higher levels correlate with worse organ dysfunction and adverse outcomes (7). The relatively elevated PCT values in patients with stage 3 AKI in our cohort are therefore consistent with the concept that systemic inflammation contributes to, and reflects, more severe kidney injury.

Taken together with existing literature, our findings support the view that AKI behaves as a systemic disorder from its earliest stages, rather than only in advanced disease. These insights frame kidney–lung crosstalk as a tightly interconnected multiorgan response network, with AKI acting as an active driver rather than a passive consequence of systemic illness.

Recognizing this interplay is therefore essential for early identification of respiratory involvement during AKI and for improving outcomes across interconnected organ systems.

Beyond the kidney–lung interaction, the potential pathogenic role of a gut–lung–kidney axis may represent a novel and promising area for future investigation. Alterations in gut permeability, immune activation, and microbial translocation may amplify systemic inflammation and distant organ dysfunction during AKI, potentially contributing to early pulmonary involvement. This axis may therefore constitute an unexplored substrate for translational research aimed at identifying early and previously underrecognized biomarkers of organ cross-talk.

From a practical standpoint, this may support a more systematic assessment of respiratory status in AKI patients, including careful clinical evaluation, judicious use of chest imaging when clinically indicated, and attention to early signs of impaired gas exchange. Furthermore, the identification of early pulmonary involvement raises the possibility that novel or underrecognized biomarkers of inflammation, complement activation, or mitochondrial dysfunction could help identify patients at higher risk for organ cross-talk before overt clinical deterioration occurs.

Overall, this study reinforces the concept of AKI as a systemic syndrome and supports a shift from an exclusively kidney-centered approach toward a broader, multi-organ perspective in the early management of AKI.

## Figures and Tables

**Table 1:** Baseline Characteristics by Pulmonary Involvement Status.

	Pulmonary Involvement 118 pts	Non Pulmonary Involvement 68 pts	*p*
AKI stage 1	20 (17%)	18 (27%)	<0.001
stage 2	24 (20%)	32 (47%)	
stage 3	74 (63%)	18 (26%)	
WBC (×10^3^/µL)	12.17 ± 6.30	6.79 ± 6.21	<0.001
CRP (mg/L)	107.8 ± 94.0	64.0 ± 59.7	<0.001
PCT (ng/mL)	8.86 ± 23.3	3.22 ± 5.84	<0.001
Pre-existing CKD	77 (65%)	25 (37%)	<0.001
Age (years)	76 ± 11	60 ± 16	<0.001

Results are expressed as Mean ± SD. AKI: Acute Kidney Injury; CKD: chronic kidney disesase; CRP: C reactive protein; PCT: procalcitonine; WBC: white blood cells.

## Data Availability

No new data were created or analyzed in this study.

## Abbreviations

The following abbreviations are used in this manuscript:

ACE	Angiotensin-Converting Enzyme
AKI	Acute Kidney Injury
ALI	Acute Lung Injury
ARDS	Acute Respiratory Distress Syndrome
ATP	Adenosine Triphosphate
COPD	Chronic Obstructive Pulmonary Disease
DAMP	Damage-Associated Molecular Pattern
ENaC	Epithelial Sodium Channel
EPO	Erythropoietin
HIF	Hypoxia-Inducible Factor
HMGB1	High Mobility Group Box 1
IL	Interleukin
IRI	Ischemia–Reperfusion Injury
MAC	Membrane Attack Complex
mTORC1	Mechanistic Target of Rapamycin Complex
NET	Neutrophil Extracellular Trap
NF-κB	Nuclear Factor Kappa B
pCO_2_	Partial Pressure of Carbon Dioxide
RAAS	Renin–Angiotensin–Aldosterone System
ROS	Reactive Oxygen Species
TLR	Toll-Like Receptor
TNF-α	Tumor Necrosis Factor Alpha
VILI	Ventilator-Induced Lung Injury
